# Novel AlN/Pt/ZnO Electrode for High Temperature SAW Sensors

**DOI:** 10.3390/ma10010069

**Published:** 2017-01-16

**Authors:** Xingpeng Liu, Bin Peng, Wanli Zhang, Jun Zhu, Xingzhao Liu, Meng Wei

**Affiliations:** State Key Laboratory of Electronic Thin Films and Integrated Devices, University of Electronic Science and Technology of China, Chengdu 610054, China; tadyliu@outlook.com (X.L.); wlzhang@uestc.edu.cn (W.Z.); junzhu@uestc.edu.cn (J.Z.); xzliu@uestc.edu.cn (X.L.); wm2lzx1314@hotmail.com (M.W.)

**Keywords:** high temperature electrode, SAW sensor, electrical resistance, langasite

## Abstract

In order to develop a film electrode for the surface acoustic wave (SAW) devices working in high temperature, harsh environments, novel AlN/Pt/ZnO multilayers were prepared using pulsed laser deposition (PLD) systems on langasite (LGS) substrates. The AlN film was used as a protective layer and the ZnO buffer layer was introduced to improve the crystal quality of Pt films. The results show that the resistances of Pt and AlN/Pt film electrodes violently increase above 600 °C and 800 °C, respectively, while the resistances of AlN/Pt/ZnO electrodes have more stable electrical resistance from room temperature to 1000 °C. The AlN/Pt/ZnO electrode, where the ZnO film was deposited at 600 °C, has the best temperature stability and can steadily work for 4 h at 1000 °C. The mechanism underlying the stable resistance of the AlN/Pt/ZnO electrode at a high temperature was investigated by analyzing the microstructure of the prepared samples. The proposed AlN/Pt/ZnO film electrode has great potential for applications in high temperature SAW sensors.

## 1. Introduction

Many efforts in recent years have been focused on surface acoustic wave (SAW) sensors [1,2], mainly due to their excellent wireless, passive, small, multifunctional quality, and their ability to be widely applied to systems sensing temperature [3], pressure [4], and strain [5] et al. With the progress of science and technology, these SAW sensors, which work in high temperature, harsh environments, are in high demand [6]. The major challenge of SAW sensors operating at high temperatures is to prepare stable high temperature film electrodes, since film electrodes such as Pt [7], Pt-Rh [8], Ir-Rh [9], Ru-Al [10], W/Mo [11], W [12], or Pt-Ni/Pt-Zr [13] film electrodes, always undergo rapid agglomeration and recrystallization above 700 °C, resulting in the discontinuity of film electrodes, an increase in resistance, and a failure of the SAW sensor. Until now, many researchers have made much effort to solve this problem. Moulzolf [8,14] co-deposited Pt/Rh (10%) as a film electrode and used HfO_2_ as a passivation coating to hinder agglomeration and recrystallization. Taguetta [9] used Ir-Rh alloy electrodes with different compositions and found that the Ir-Rh film electrodes with optimum composition ratio can tolerate temperatures as high as 800 °C. Rane [12] used tungsten film electrodes to prevent the diffusion of Ga and O atoms from the substrate, into the tungsten film, and obtained stable electrodes up to 800 °C. Seifert [15] prepared RuAl film electrodes with a SiO_2_ barrier, which was able to work stably at 800 °C for 10 h. However, the SAW electrode was unable to steadily work above 1000 °C because the recrystallization phenomenon of the electrode film was difficult to prevent.

From the previous reports, it can be seen that the agglomeration and recrystallization of metal electrodes always occur at a high temperature. We think that one of the reasons underlying this is that the as-deposited metal film electrode is not crystallized fully, or that the crystal quality of the metal film electrode is poor. Because of this, the metal film electrode recrystallizes at a high temperature and its resistance increases dramatically. Therefore, we would predict that the stability of a film electrode at a high temperature can be improved if the as-deposited film electrode is already well-grown, with a good crystal quality. In this work, we deposited Pt film electrodes on Langasite (LGS) piezoelectric substrate, because LGS substrate can work at high temperatures [16]. The LGS substrates which we used are inclined and cut with an irregular orientation, so the Pt film electrode deposited directly on them doesn’t have a good crystal structure. To solve this problem, we chose ZnO as a buffer layer because the use of a ZnO film is a very easy way of forming an orientation texture [17,18]. Besides, ZnO film was introduced as a buffer layer for Pt film electrodes because the lattice mismatch between Pt and ZnO is small, and the ZnO buffer layer is then able to prompt the crystallization and preferential (111) orientation growth of the Pt film [19]. Finally, an AlN capping layer was deposited on the Pt film and acted as a passive coating. The characteristics of the proposed AlN/Pt/ZnO film electrodes, from room temperature to 1000 °C, have been explored.

## 2. Results and Discussion

**Figure 1:**
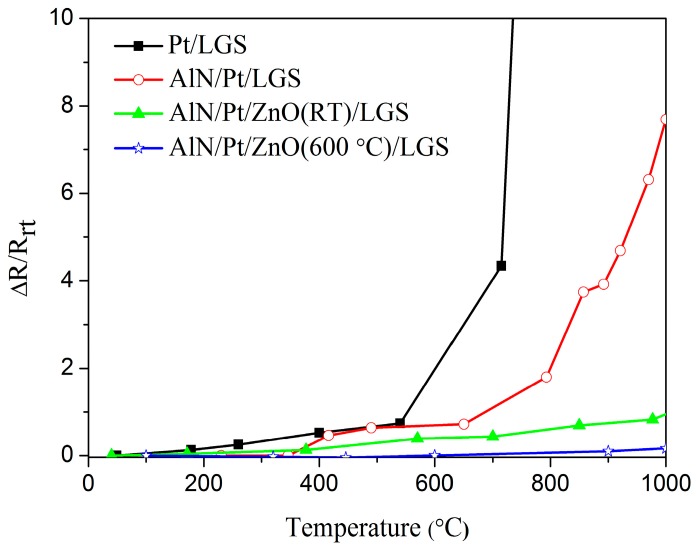
Relative resistance change for different samples as a function of temperature. The AlN/Pt/ZnO(RT)/LGS and AlN/Pt/ZnO(600 °C)/LGS samples correspond to the AlN/Pt/ZnO/LGS samples where the ZnO film was deposited at room temperature and 600 °C, respectively.

Figure 1 shows the real-time, relative resistance change (ΔR/R_rt_) as a function of temperature for Pt/LGS, AlN/Pt/LGS, AlN/Pt/ZnO(RT)/LGS, AlN/Pt/ZnO(600 °C)/LGS samples, where R_rt_ is the resistance of the sample at room temperature and ΔR is the difference between the resistances at high temperature and room temperature. It can be seen that the resistance of the Pt/LGS sample sharply increases above 600 °C, and increases by 22 times when it is heated to 800 °C, from room temperature. The resistance of the AlN/Pt/LGS sample increases slowly below 800 °C and increases by eight times when it is heated to 1000 °C, from room temperature. It can be seen that the resistance of the AlN/Pt/LGS electrode continues to increase at a remarkable rate, though the AlN capping layer can improve the stability of the Pt film electrode to some extent, by comparing the resistance of Pt/LGS and of AlN/Pt/LGS samples. It is interesting to note that the resistance of the AlN/Pt/ZnO(RT)/LGS sample changes very slowly between room temperature and 1000 °C. The relative resistance change of the AlN/Pt/ZnO(RT)/LGS sample is about 82% at 1000 °C, which indicates that the ZnO buffer layer plays a prominent role in hindering the degradation of Pt film electrodes. Furthermore, it can be observed that the resistance of the AlN/Pt/ZnO(600 °C)/LGS sample is more stable than that of the AlN/Pt/ZnO(RT)/LGS sample. The relative resistance change of the AlN/Pt/ZnO(600 °C)/LGS sample is only about 17% at 1000 °C, which means that this AlN/Pt/ZnO(600 °C)/LGS electrode is stable enough to work at room temperature and 1000 °C.

**Figure 2:**
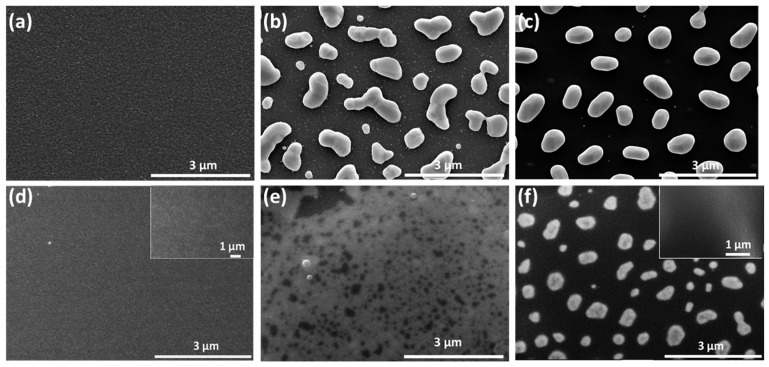
Surface topography of Pt/LGS samples (**a**) before high temperature measurement; and (**b**) after 800 °C resistance measurement; and (**c**) after 1000 °C resistance measurement; Surface topography of AlN/Pt/LGS samples (**d**) before high temperature measurement; and (**e**) after 800 °C resistance measurement; and (**f**) after 1000 °C resistance measurement. Insets shown in (d); and (f) present the surface topography of AlN/LGS samples before and after 1000 °C resistance measurement, respectively.

When studying Figure 1, it is clear to view that the resistance changes of these four samples vary. The resistance change of the metal film electrode is dependent on its microstructure. In order to investigate the changes in these samples, before and after being exposed to a high temperature, microstructural analyses were performed by using SEM and XRD. Figure 2 shows the surface topography of Pt/LGS and AlN/Pt/LGS samples before and after high temperature resistance measurements have been recorded. When looking at Figure 2a, it can be observed that the surface topography of the Pt/LGS sample before measurement is smooth, showcasing no big grains. However, after the 800 °C and 1000 °C resistance measurement, completely isolated grains appear at the surface of the Pt/LGS sample, as shown in Figure 2b,c, indicating that the Pt film experiences agglomeration and recrystallization above 800 °C. As can be seen in Figure 2c, the grain size is about 1 μm. As a result of this, the Pt film becomes discontinuous and loses its electrical conductivity at high temperatures. Similarly, we can view that the AlN/Pt/LGS sample also exhibits a smooth surface without obvious big grains before measurements are taken, as shown in Figure 2d. After the 800 °C resistance measurement, some discontinuities appear on the surface of the AlN/Pt/LGS sample, as shown in Figure 2e, indicating that the agglomeration and recrystallization is partly prevented. This result demonstrates the increasingly stable electrical conductivity of the AlN/Pt/LGS sample than the Pt/LGS sample above 800 °C, as shown in Figure 1. Additionally, the surface topography of the AlN/Pt/LGS sample after the 1000 °C resistance measurement is shown in Figure 2f which reveals completely isolated grains with a smaller size (500 nm), leading to a sharp decrease in electrical conductivity above 800 °C. The insets in Figure 2d,f show the surface topography of the AlN/LGS sample before and after the 1000 °C resistance measurement, respectively. No obvious grains are observed after this measurement, which indicates that the appearance of big grains in the AlN/Pt/LGS samples derive from the Pt film rather than the AlN film.

**Figure 3:**
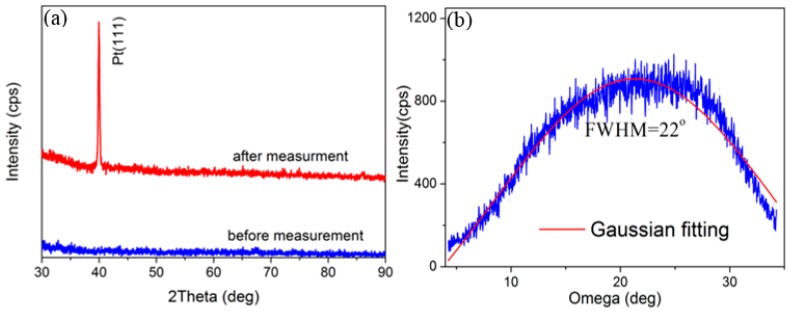
(**a**) θ–2θ scans of AlN/Pt/LGS samples before and after high temperature measurement; (**b**) Rocking curve of Pt (111) of AlN/Pt/LGS samples after high temperature measurement.

Figure 3 shows the θ–2θ XRD scan and omega scan of the AlN/Pt/LGS sample, both before and after the 1000 °C resistance measurement. No obvious peak can be identified from the XRD curve before measurement. However, a clear Pt (111) peak appears after the 1000 °C resistance measurement. These results suggest that either the Pt film is not crystalized, or that the crystalline quality is poor before measurement, and that the Pt film only crystallized after the high temperature measurement. It can thus be concluded that the Pt phase witnessed great recrystallization and formed completely isolated grains, leading to the remarkable increase in resistance. The FWHM of the Pt (111), characterized by the ω scan as shown in Figure 3b, was as large as 22°, indicating that the crystal quality isn’t good enough. At the same time, no AlN peaks were identified in Figure 3a. We think that this may be due to the low precision of our X-ray diffraction and the low thickness of our AlN top layer. In general, only the AlN films with a thickness of a several hundred nanometers can show obvious AlN peaks [20,21,22]. However, the thickness of the AlN film in our work is only about 30 nm.

**Figure 4:**
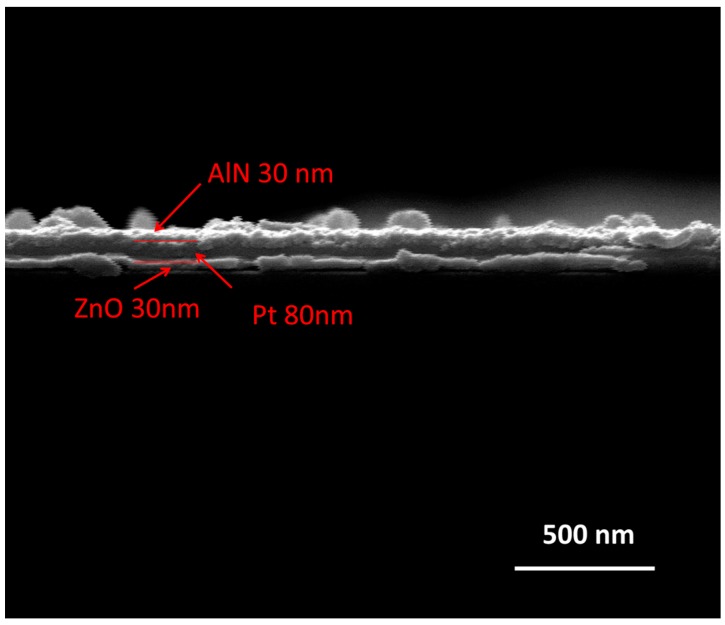
SEM image of the cross-section of the AlN/Pt/ZnO(RT)/LGS sample.

**Figure 5:**
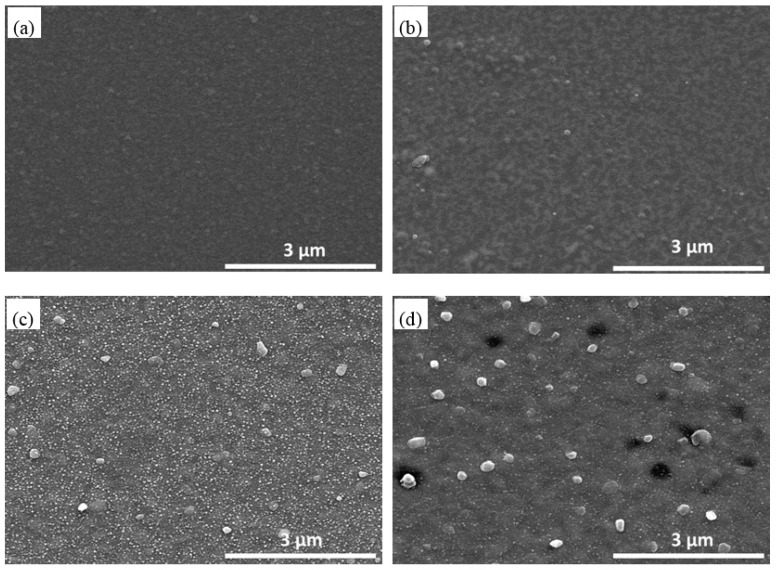
Surface topography of AlN/Pt/ZnO(RT)/LGS samples (**a**) before; and (**b**) after 1000 °C resistance measurement. Surface topography of AlN/Pt/ZnO(600 °C)/LGS samples; (**c**) before; and (**d**) after 1000 °C resistance measurement.

From the microstructure evolution of the AlN/Pt/LGS sample before and after high temperature, we expect that the stability of the Pt film at high temperature can be improved, if we can deposit a Pt film with good crystalline quality. Next, we discuss the effects of the ZnO buffer layer on the microstructure of the Pt film at high temperature. The cross-sectional SEM image of AlN/Pt/ZnO(RT)/LGS is shown in Figure 4. From this SEM result, we can clearly observe that there are three film layers, representing ZnO, Pt, and AlN films, respectively. The surface topography of the AlN/Pt/ZnO(RT)/LGS sample and the AlN/Pt/ZnO(600 °C)/LGS sample, before and after the 1000 °C measurement, are shown in Figure 5.

For AlN/Pt/ZnO(RT)/LGS samples, the surface topography before the 1000 °C measurement is shown in Figure 5a. The surface is smooth and there are no obvious grains. However, after the high temperature measurement, we can observe many discontinuities and small grains in Figure 5b. These are different to the completely isolated Pt grains in Figure 2c, and are small, without being completely isolated. Because of this, the resistance of the AlN/Pt/ZnO(RT)/LGS sample after the high temperature measurement only increased slightly. A comparison of the surface topography of AlN/Pt/ZnO(600 °C) samples before and after the high temperature measurement are shown in Figure 5c,d. Figure 5c presents many extreme small grains and few big grains. These small grains are considered to be AlN particles. Following the high temperature measurement, no obvious isolated grains appeared, thus leading to a stable electrical conductivity, partly explaining the horizontal straight curve of AlN/Pt/ZnO(600 °C)/LGS shown in Figure 1. Furthermore, a high number of small grains disappeared after the high temperature measurement, indicating that the AlN phase recrystallized during the high temperature measurement and formed a big crystal.

**Figure 6:**
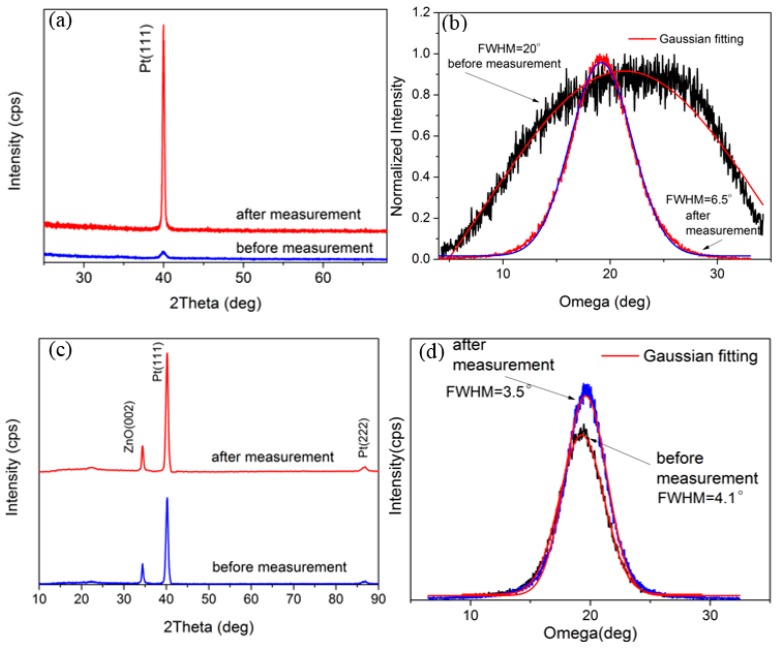
(**a**) θ–2θ scan; and (**b**) rocking curves of the AlN/Pt/ZnO(RT)/LGS samples before and after 1000 °C resistance measurement; (**c**) θ–2θ scan; and (**d**) rocking curves of the AlN/Pt/ZnO(600 °C)/LGS samples before and after 1000 °C resistance measurement.

Figure 6 shows the θ–2θ scan and omega scan of the AlN/Pt/ZnO(RT)/LGS samples and the AlN/Pt/ZnO(600 °C)/LGS samples before and after the high temperature measurement. When studying Figure 6a, we can see that the Pt (111) peak with low intensity appears before the measurement has been taken. The low intensity, and the large FWHM shown in Figure 6b, indicate that the Pt phase formed a preferential (111) orientation with poor crystal quality when it was deposited at room temperature. After the 1000 °C resistance measurement, the intensity of the Pt (111) peak violently increased, and the FWHM decreased from 20° to 6.5°, indicating that the Pt phase was still recrystallized at high temperature. The pre-existing preferential Pt (111) orientation is considered to play an important role in partly weakening the recrystallization at high temperature, and thus the AlN/Pt/ZnO(RT) electrode films obtained more stable electrical conductivity at a high temperature than the AlN/Pt electrode. As a comparison, the sample deposited at 600 °C presents a Pt (111) peak with high intensity and small FWHM before the 1000 °C resistance measurement, as shown in Figure 6c, indicating that the Pt film has good crystal quality before the high temperature measurement. After this measurement, both the intensity of the Pt (111) peak and the FWHM almost remained the same, and it shows that the recrystallization was almost totally prevented due to the pre-existing good crystal quality.

**Figure 7:**
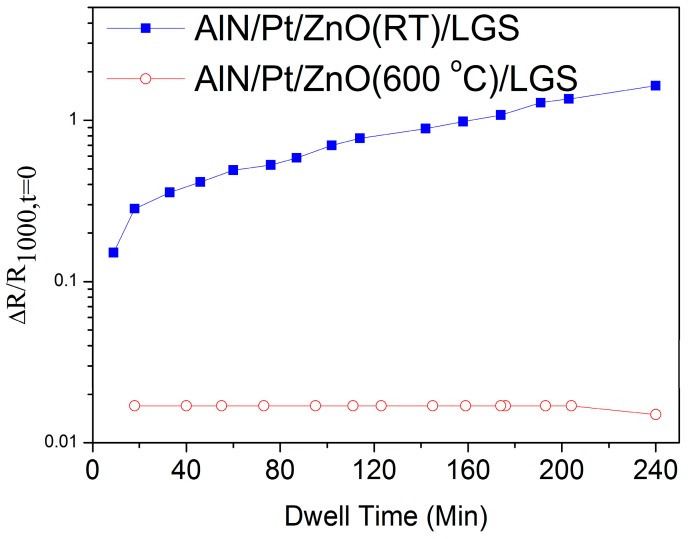
Relative resistance change of AlN/Pt/ZnO(RT)/LGS and AlN/Pt/ZnO(600 °C)/LGS samples as a function of time at 1000 °C for 4 h.

Figure 7 shows the real-time relative resistance measurement of AlN/Pt/ZnO(RT)/LGS and AlN/Pt/ZnO(600 °C)/LGS samples at 1000 °C for 4 h, where R_1000,t=0_ represents the value of resistance at the beginning of the measurement at 1000 °C. During the 4 h heat preservation process, the resistance of the AlN/Pt/ZnO(RT)/LGS sample slowly increased by 1.6 times and the resistance of the AlN/Pt/ZnO(600 °C)/LGS sample remained the same. Because the Pt film became more isolated, as shown in Figure 5b, the electronic transmission paths of the AlN/Pt/ZnO(RT) electrode became fewer and more crowded, resulting in a loss in conductivity. As a comparison, the resistance of the AlN/Pt/ZnO(600 °C)/LGS sample didn’t increase during the preservation process at 1000 °C, due to the more stable structrue of the Pt phase at 1000 °C, which derived from better crystal quality. In this case, the Pt film has better continuity, resulting in a better stability of the electric conductivity at a high temperature. Compared to other multi-layer-films losing part of their electrical conductivity after annealing at 1000 °C for 4 h, like Pt-Ni multilayer films [13], these AlN/Pt/ZnO(600 °C) electrode films present more stability in their electrical conductivity after the 1000 °C resistance measurement for 4 h, because of the pre-existing good crystal quality of the Pt (111) phase.

## 3. Materials and Methods

All films were prepared in a pulsed laser MBE system, using a KrF (λ = 248 nm) excimer laser (lambda physic, Goettingen, Germany). Highly purified AlN, Pt, and ZnO targets were used in this work and the distance from the target to the substrate was 5 cm. The AlN/Pt/ZnO electrodes were deposited on LGS piezoelectric substrate, cut with Euler angle of (0°, 138.5° and 116.6°). The thickness of AlN, Pt, and ZnO layers were 30, 80, and 30 nm, respectively. Before being deposited, the LGS substrates were ultrasonically cleaned in anhydrous alcohol for 6 min, followed by a N_2_ drying process. Then, the substrates were put into the deposition chamber. During the entire depositing process of AlN/Pt/ZnO samples, the base pressure was set at 5 × 10^−5^ Pa. ZnO was directly deposited on the LGS substrate as a buffer layer for 30 min. The substrate temperature was kept at room temperature or 600 °C during depositing ZnO films. The samples were named as AlN/Pt/ZnO (RT) and AlN/Pt/ZnO(600 °C), respectively. The laser energy density was about 4 J·cm^−2^ at a frequency of 2 Hz (30 nm). Next, the Pt film was deposited on the ZnO buffer layer, with a laser energy of 6 J·cm^−2^ at a frequency of 1 Hz for 1 h (80 nm). Finally, the AlN capping layer was deposited on the Pt films for 20 min, with a laser energy of 4 J·cm^−2^ at a frequency of 2 Hz (30 nm). In order to better understand the role of the ZnO buffer layer, Pt and AlN/Pt electrodes without ZnO buffer layer were also deposited on LGS substrates at room temperature, with the same thickness and the same depositing conditions.

The resistances of the samples were measured from room temperature to 1000 °C in air, within a tube furnace with a resistance meter (Keithley 2400, Microlease, Cary, NC, USA). The heating rate was 4 °C per minute. After the high temperature resistance measurement had been taken, the samples were cooled down to room temperature in a natural cooling condition. Film crystalline structure and texture were measured by X-ray diffraction (D1, Bede X-ray Metrology, Durham, UK), both before and after the high temperature resistance measurement. An omega scan and θ–2θ scan were both performed in order to precisely characterize the orientation of the metal film electrodes. A scanning electron microscope (JSM-7500F, JEOL, Peabody, MA, USA) was used to characterize the surface topography of the samples, both before and after the high temperature resistance measurement.

## 4. Conclusions

In this work, ZnO film is introduced as a buffer layer in order to improve the crystal quality of the Pt film, so that the recrystallization of the Pt phase is hindered at a high temperature. In this way, the stability of the electrical resistance of the novel AlN/Pt/ZnO multilayer films electrode, at is greatly improved when at 1000 °C. It is found that the AlN capping layer can improve the stability of the Pt film electrode, to some extent. The ZnO buffer layer can prompt the preferential (111) orientation growth of Pt films, thereby hindering the recrystallization in Pt films with great (111) preferred orientation. This is especially the case for the AlN/Pt/ZnO(600 °C) electrode, which can steadily work at 1000 °C for at least 4 h. It suggests that the recrystallization of Pt films is dependent on their crystal quality. The proposed AlN/Pt/ZnO electrode structure has great potential for applications not only in SAW sensors, but also in other sensors which work in high temperature, harsh environments.
